# Assessing Recent Smoking Status by Measuring Exhaled Carbon Monoxide Levels

**DOI:** 10.1371/journal.pone.0028864

**Published:** 2011-12-16

**Authors:** AnnSofi Sandberg, C. Magnus Sköld, Johan Grunewald, Anders Eklund, Åsa M. Wheelock

**Affiliations:** 1 Science for Life Laboratory, Department of Oncology-Pathology, Karolinska Institutet, Stockholm, Sweden; 2 Division of Respiratory Medicine, Department of Medicine, Karolinska Institutet and Karolinska University Hospital, Stockholm, Sweden; University of Pittsburgh, United States of America

## Abstract

**Background:**

Cigarette smoke causes both acute and chronic changes of the immune system. Excluding recent smoking is therefore important in clinical studies with chronic inflammation as primary focus. In this context, it is common to ask the study subjects to refrain from smoking within a certain time frame prior to sampling. The duration of the smoking cessation is typically from midnight the evening before, i.e. 8 hours from sampling. As it has been shown that a proportion of current smokers underestimates or denies smoking, objective assessment of recent smoking status is of great importance. Our aim was to extend the use of exhaled carbon monoxide (CO_breath_), a well-established method for separating smokers from non-smokers, to assessment of recent smoking status.

**Methods and Findings:**

The time course of CO_breath_ decline was investigated by hourly measurements during one day on non-symptomatic smokers and non-smokers (6+7), as well as by measurements on three separate occasions on non-smokers (n = 29), smokers with normal lung function (n = 38) and smokers with chronic obstructive pulmonary disease (n = 19) participating in a clinical study. We used regression analysis to model the decay, and receiver operator characteristics analysis for evaluation of model performance. The decline was described as a mono-exponential decay (r^2^ = 0.7) with a half-life of 4.5 hours. CO decline rate depends on initial CO levels, and by necessity a generic cut-off is therefore crude as initial CO_breath_ varies a lot between individuals. However, a cut-off level of 12 ppm could classify recent smokers from smokers having refrained from smoking during the past 8 hours with a specificity of 94% and a sensitivity of 90%.

**Conclusions:**

We hereby describe a method for classifying recent smokers from smokers having refrained from smoking for >8 hours that is easy to implement in a clinical setting.

## Introduction

Smoking is a major factor in heart disease, stroke and chronic lung disease, and the association of smoking with altered levels of inflammatory markers is well documented [1], [2], [3]. It is known that inflammatory markers have a temporal relationship to smoking [4], [5], [6], and that the acute effects of cigarette smoke have an impact on a number of cellular and biochemical measures in the lung [7], [8]. Thus, in studies focusing on chronic inflammation of the lung, such as mechanistic investigations of chronic obstructive pulmonary disease (COPD) and rheumatoid arthritis, the acute inflammatory effects of smoking is a confounding factor. In this context it is common to ask the study subjects to refrain from smoking within a certain time frame prior to sampling. The duration of the smoking cessation is typically from midnight the evening before, i.e. no smoking within 8 hours from sampling. However, as it has been shown that a proportion of current smokers underestimates or denies smoking [9], [10], the ability to objectively assess recent smoking status is of great importance.

Objective measures of smoking status include cotinine levels in urine, however the half-life of cotinine is 17 hours [11] and hence more suitable for distinguish smokers from non-smokers, not to assess recent smoking status among smokers [12]. Measuring carbon monoxide in exhaled breath (CO_breath_) is an immediate, non-invasive and well-established method used to classify smokers from non-smokers [13], [14]. As a constituent of cigarette smoke, carbon monoxide enters the circulation during smoking and forms carboxyhemoglobin (COHb). The elimination of CO is primarily by respiration thus there is a strong correlation between CO_breath_ and COHb [10], [13], [15] making it a useful tool for assessing smoking status. Depending on factors such as gender and physical activity [16], COHb half-life is 5–6 hours [15], [17] and is thus more suitable for estimating short term smoking abstinence. Moreover, CO_breath_ is correlated to the number of cigarettes smoked during the past 24 hours [18], [19], [20] as well as to the time since last cigarette smoked [19].

A number of cut-off levels ranging from 5–6 ppm depending on study population have been suggested for classification of smokers from non-smokers [19], [20], [21], [22]. At present, there is however no method for using CO_breath_ to assess recent smoking status among smokers. In this study, we have investigated whether exhaled carbon monoxide can be used as a tool to discriminate between short term abstinence and continued smoking. Our aim was to establish a cut off value for CO_breath_ to be used for discriminating recent smokers from smokers having refrained from smoking for at least 8 hours.

## Materials and Methods

### Investigating CO_breath_ Decline: Subjects and Study Design

#### Group 1: Model group

A training set of 13 individuals was used for modelling of CO_breath_ decline over an 8 hour period. The model group consisted of 6 non-symptomatic current smokers and 7 non-smokers aged 45–66 years (Table 1). They had no self-reported airway symptoms and were not taking any medication. The subjects were allowed to smoke at one occasion in the morning, and thereafter the CO_breath_ levels were measured hourly throughout the day as the subjects refrained from smoking. Baseline CO_breath_ levels measured prior to smoking as well as CO_breath_ levels measured immediately after smoking were also assessed. The participants were instructed on how to use the portable CO monitor and then performed measurements on their own (see below).

**Table 1:** Characteristics for non-smokers and smokers (group 1).

Variable	Non-smokers (n = 7)	Smokers (n = 6)
**Age**	60 (48–66)	56 (45–65)
**Sex**		
**Female**	4	3
**Male**	3	3
**Pack years**	0	27.5 (22–34)
**Cig/day past 6 months.**	0	15 (10–20)

Values are given as median (range). Pack years = (number cigarettes smoked per day)/(content of 1 packet of cigarettes (20)) * years as a smoker.

#### Group 2: Test group

A test set of 86 individuals was used to evaluate the model constructed from the training set. The test group were participants of a clinical study at the Karolinska University Hospital Solna, Sweden, (Table 2) and consisted of healthy non-smokers (n = 29), current smokers with normal lung function (n = 38), and current smokers with COPD of GOLD stage I and II (mild to moderate disease) (n = 19) [23]. In COPD patients, the ratio FEV_1_/FVC (FEV_1_: forced expiratory volume in 1 second; FVC: forced vital capacity) was <0.7 and FEV_1_ was 50–100% of predicted after inhalation of two doses of terbutaline à 0.5 mg (Bricanyl Turbuhaler®, AstraZeneca AB, Södertälje, Sweden). All smokers of group 1 and 2 had a smoking history of >10 pack years and a current cigarette consumption of >10 cigarettes/day. The COPD patients had not undergone any oral or inhaled corticosteroid treatment for the last 3 months and had not experienced any signs of disease worsening (exacerbation) within the past 3 months. In addition, in vitro screening for the presence of specific IgE antibodies against common inhaled allergens (Phadiatop®, Pharmacia-Upjohn, Uppsala, Sweden) was negative in all participants. CO_breath_ measurements were assisted by a research nurse at scheduled visits at the clinic (3–4 separate occasions/individual), at which point the study subjects were asked to estimate time since last cigarette smoked (see below). Body mass index (BMI), blood haemoglobin and high sensitive C-reactive protein (CRP) level were determined during visits.

**Table 2:** Characteristics and lung function data for non-smokers, smokers with normal lung function and smokers with COPD (group 2).

Variable	Non-smokers with normal lung function (n = 29)	Smokers with normal lung function (n = 38)	Smokers with COPD (n = 19)
**Age**	59 (46–66)	52 (44–65)	57 (47–62)
**Sex**			
**Female**	15	20	9
**Male**	14	18	10
**Pack years #**	0	34 (15–49)	42 (23–62)
**Cig/day past 6 months.**	0	20 (10–40)	20 (2.5–25)
**FEV1, % of predicted #¤**	119 (89–141)	106.5 (91–140)	52 (51–97)
**FEV1/FVC # ¤**	0.82 (0.70–0.91)	0.78 (0.71–0.88)	0.61 (0.45–0.69)
DL_CO *_ ¤	92 (74–116)	80 (48–106)	68 (50–81)

Values are given as median (range). Statistically significant differences (p<0.05) between groups are indicated * (Non-smokers *vs* Smokers), # (Smokers with COPD *vs* Smokers) and ¤ (Smokers with COPD *vs* Non-smokers).

FEV_1_: Forced expiratory volume in 1 second, measured post-bronchodilator.

FVC: Forced vital capacity, measured post-bronchodilator.

DL_CO_: Carbon monoxide diffusion capacity.

### Ethics Statement

The study was approved by the local ethical board (Stockholm, Sweden; ethical committee diary number 2006/959-31/1) and performed in accordance with the Declaration of Helsinki. Informed, written consent was obtained from all study participants.

### Measurement of Exhaled CO (CO_breath_)

CO_breath_ levels were measured in triplicate at each time point using the Smokerlyzer® Micro EC50 device (Bedfont Scientific Ltd, Kent, U.K.) according the manufacturer's recommendations. In brief, subjects were asked to hold their breath for 20 seconds to allow COHb to form equilibrium with alveolar CO. The subjects then exhaled slowly and fully into the mouthpiece of the instrument during which CO_breath_ was recorded. The CO_breath_ levels are given in parts per million (ppm). The device was calibrated according to the manufacturer's instructions prior to use, and then biannually throughout the study.

### Statistical Analyses and Modelling

Statistical analysis was performed using GraphPad Prism version 5.02 (GraphPad Software Inc., USA). Mean of triplicate measurements, standard deviation (SD) and coefficient of variance (CV) were calculated for each time point. Differences between 2 groups were investigated using Mann-Whitney test. Comparisons between 3 groups were calculated using Kruskal-Wallis analysis of variance followed by Dunn's post-test. Correlations were calculated according to Spearman's rank correlation (p <0.05 was considered statistically significant).

To investigate CO decline over time, nonlinear regression was used on the raw data from group 1. A mono-exponential equation [Y = Y0*exp(−K*x)], where Y = ppm CO_breath_ at time x hours since smoking, Y0 = CO_breath_ immediately after smoking (i.e. at x = 0) and x = time elapsed since last cigarette was used to model the decay. The validity of the mono-exponential equation was tested by plotting the natural logarithm of CO_breath_ (ln(CO_breath_)) versus time followed by a linearity test using linear regression analysis [24]. CO_breath_ half-life was calculated as ln(2)/slope. Prediction limits (95%) for the model were calculated and evaluated as cut-offs. To evaluate the robustness of the model and the generated cut-off, cross validation was performed by dividing the non-symptomatic smokers in group 2 into six cross-validation sets, each consisting of a randomly selected training set (n = 30) and test set (n = 8).

Receiver-operator characteristics (ROC) analysis for CO_breath_ was performed using GraphPad Prism version 5.02 and Excel (Microsoft, Redmond, OR, USA), and sensitivity and specificity levels were calculated. To evaluate the robustness of the ROC analysis, the analysis was performed 6 times and each time 8 randomly selected individuals were excluded from the analysis.

## Results

### Exhaled CO in Smokers versus Non-smokers

**Figure 1:**
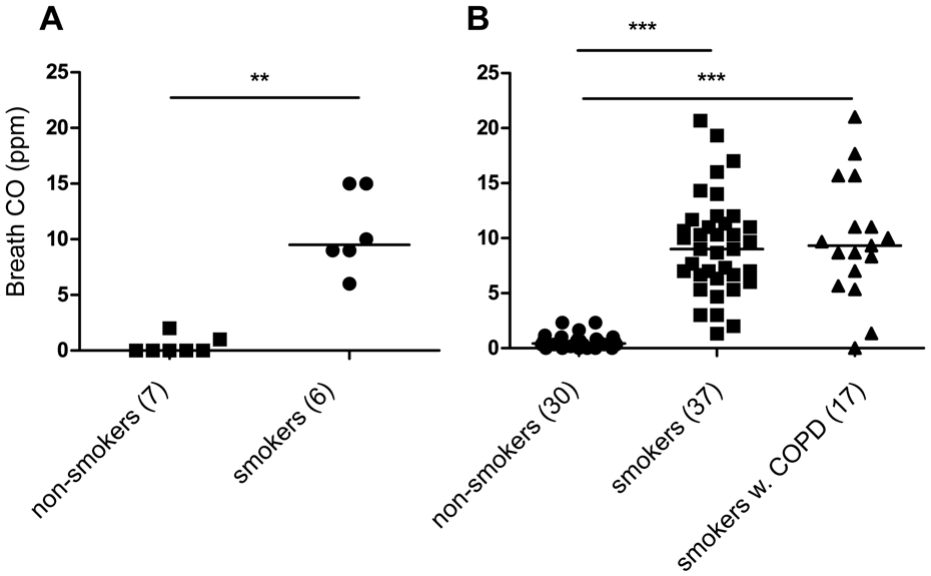
Baseline CO_breath_ levels measured on non-smokers, smokers with normal lung function and smokers with COPD. **A.** CO_breath_ from smokers and non-smokers recruited for a time course study of CO_breath_ decline (group 1). CO_breath_ (ppm) was measured in the morning; smokers having refrained from smoking during the past >8 hours. ** indicates p<0.01. **B.** CO_breath_ measured on smokers with normal lung function (“smokers”), smokers with COPD and non-smokers with normal lung function (group 2). CO_breath_ (ppm) was measured in the morning; smokers having refrained from smoking during the past 8 hours. *** indicates p<0.001.

In line with previous findings, CO_breath_ levels were significantly higher in smokers (>8 hours after last cigarette) than in non-smokers in both study groups (Figure 1A and B). No significant difference in breath CO was observed comparing smokers with normal lung function and smokers with COPD, GOLD stage I–II (Figure 1B). The CV_median_ calculated from the triplicate measurements was 8.8% and 10.2% for non-symptomatic smokers and smokers with COPD, respectively. The high relative variance for non-smokers (CV_median_ = 150%) was explained by the overall low absolute values (0–3 ppm) close to the detection limit.

### Time Course of CO_breath_ Decline

#### Group 1

**Figure 2:**
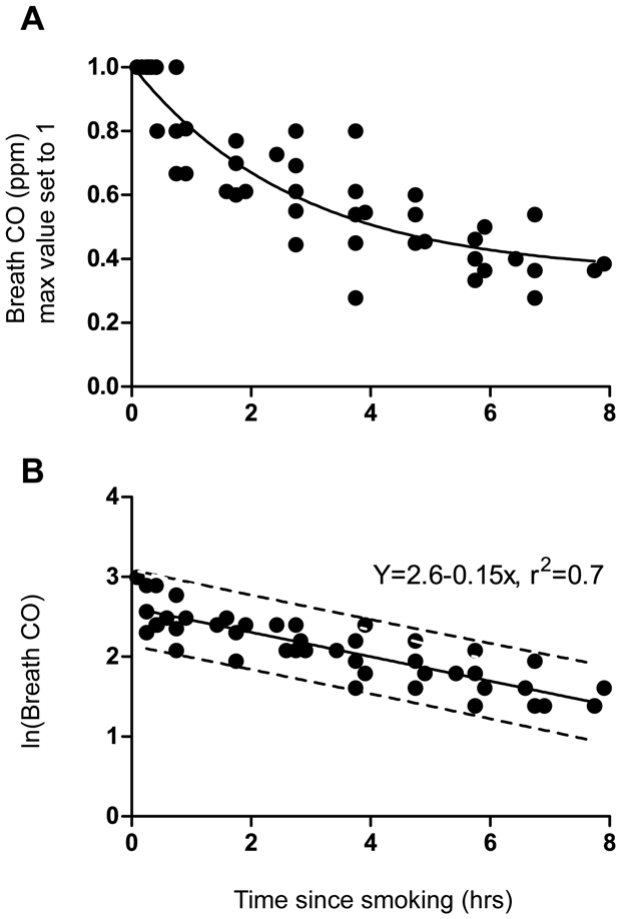
Time course of CO_breath_ decline after smoking one cigarette (group 1). **A.** After normalisation against each individual peak value, relative CO_breath_ values were plotted as a function of time since smoking. By non-linear regression, a one phase exponential model was fitted to the decay (Y = (Y0-0.35)*e^−0.36×^+0.35, r^2^ = 0.77). **B.** The natural logarithm of CO_breath_, ln(CO_breath_) was plotted versus time since smoking, and the decay was described by linear regression (Y = Y0-0.15×, r^2^ = 0.70). From the slope, CO_breath_ half-life during the day was estimated to 4.5 hours (ln(2)/slope). The 95% prediction limits are also showed in the figure (dashed lines).

CO_breath_ measurements from smoking subjects (n = 6) were plotted versus time since smoking. The CO_breath_ levels measured in the morning 8 hours since smoking were higher than those measured in the afternoon 8 hours since smoking. For all smokers, CO_breath_ levels measured in the morning exceeded 6 ppm. Non-smokers (n = 7) had CO_breath_ levels below 3 ppm regardless of the time-of-day, with slightly elevated CO_breath_ at lunchtime (Figure S1). In smokers, CO_breath_ decline could be described as a mono-exponential decay (Y = (Y0-0.35)*e^−0.36×^+0.35; r^2^ = 0.77, Figure 2A). Logarithmic values, ln(CO_breath_), were plotted versus time since smoking (Figure 2B). Linear regression on the time course from each individual subject gave r^2^ values 0.5; 0.8; 0.8; 0.9; 0.9 and 0.9, respectively. The differences in decline rate were not statistically significant (p = 0.2), but the difference between the y-intercepts (initial CO_breath_ levels) were (p<0.0001). Given that the decline rates were similar, CO half-life was determined from the merged linear regression of all subjects (Y = 2.6-0.153×, r^2^ = 0.7) giving a CO_breath_ half-life of 4.5 hours.

#### Normalisation

CO_breath_ values measured in the morning prior to smoking were found to be higher compared to those measured at the corresponding time point after smoking in the afternoon. The magnitude of the difference was determined to be a factor of 1.33 in group 1 (Figure S1B). A likely reason for the observed difference is differences in ventilation rate during the day compared to the night (see Discussion). Under the assumption that the CO_breath_ decline for group 2 corresponded to that of group 1, the normalization factor of 1.33 was used to adjust for diurnal differences in decline rate also in group 2.

#### Group 2

No significant differences in CO_breath_ decline pattern was detected between non-symptomatic smokers and smokers with COPD. As the self-reported time since smoking varied between individuals, the recorded data was merged into 4 intervals; 0.25±0.25, 1±0.5, 9±1 and 16±2 hours since smoking (Figure S2). The differences between these time-bins in terms of gender were evaluated. CO_breath_ levels from both men and women declined significantly from 1±0.5 to 9±1 hours (p<0.0001). The CO_breath_ decline from 0.25±0.25 to 1±0.5 hours was however not large enough to be statistically significant. At time bin 9±1 (measurement performed in the morning after having refrained from smoking during the night), women had significantly lower CO_breath_ compared to men (p<0.05). No differences in exact time since smoking or differences in current cigarette consumption could explain these observations. To further investigate CO_breath_ decline over time, ln(CO_breath_) was plotted against time since smoking. When calculated separately, CO_breath_ half-life differed between men and women (9.5 and 7.2 hrs, respectively) within the time interval 0–18 hrs. Given that the differences in decline rate were non-significant, CO_breath_ data from men and women were merged in subsequent calculations.

**Figure 3:**
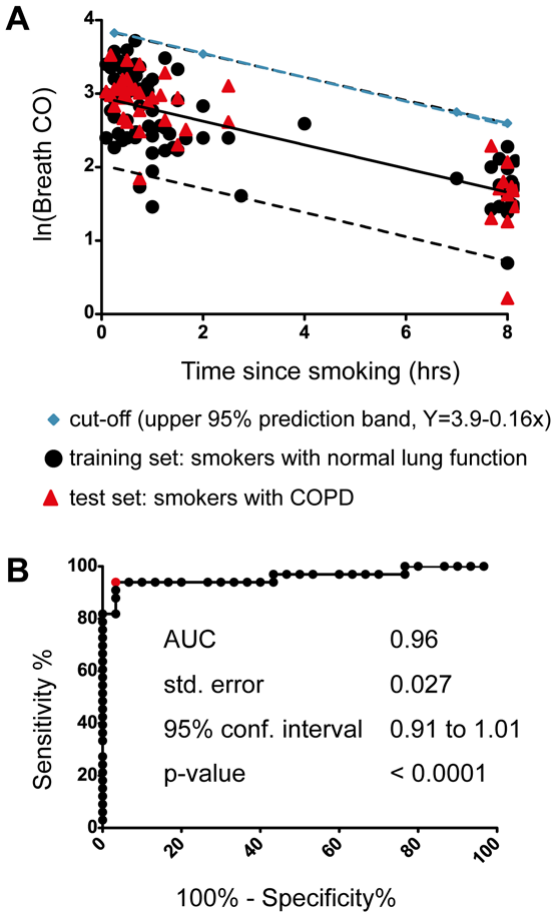
Proposed model for CO_breath_ decline (group 2), and receiver operator characteristics (ROC) analysis for evaluation of model classification performance on smoking subjects with and without COPD. **A.** CO_breath_ measurements (smokers) were recorded in the morning. After normalisation ln(CO_breath_) was plotted versus self-reported time since smoking. To allow comparisons with group 1, measurements performed >8 hours since smoking were omitted. CO decline was modelled by linear regression (solid line) on smokers with normal lung function. From the slope, CO_breath_ half-life was estimated to 4.3 hours (ln(2)/slope). The 95% prediction limits are indicated in the graph as dashed lines. The upper prediction limit (Y = 3.9-0.16×) was evaluated as a cut-off. As test set, CO_breath_ measured on smokers with COPD (n = 19) were included (indicated with triangles). **B.** CO_breath_ values measured ≤7 hours were used to estimate specificity (classified as positive for recent smoking status), and values measured between 8–10 hours since smoking were used to estimate the sensitivity (classified as negative for recent smoking status). A cut-off of 12 ppm gave a sensitivity of 94% and a median specificity of 97%. Area under the curve (AUC) was 0.96. X-axis: False positive Rate, Y-axis: True positive Rate.

As described under “Normalisation”, CO_breath_ measurements performed in the morning on smokers in group 2 were adjusted by dividing with a factor 1.33. This gave a constructed decline rate corresponding to that of group 1. The robustness of the constructed decline rate was tested by a six-fold randomized cross validation within group 2 and showed similar decline rates in all rounds. The average slope of the decline and upper 95% prediction limit (Y = 3.9-0.16×) are shown in Figure 3A. From the constructed decline rate, CO_breath_ half-life was estimated to 4.3 hours. The log-transformed decay model (ln(CO_breath_) of normalized values was further evaluated using the subgroup of smokers diagnosed with COPD (n = 19) as a test set. No significant difference between smokers and COPD patients were detected, neither in terms of slopes nor intercepts.

### Smoking History, Age and Lung Function

Smokers with normal lung function: there was a weak correlation between CO_breath_ and the number of cigarettes smoked per day during the past 6 months, but not to the cumulative smoking history in pack years or between CO_breath_ at 8 hours since smoking and absolute total lung capacity (TLC), Figure S3. No correlations were found between CO_breath_ at 8 hours since smoking and age, FEV1 (absolute or percent of predicted), TLC% of predicted, body mass index (BMI), blood haemoglobin or CRP levels. No significant correlations to the above parameters were detected for smokers with COPD.

### Model Performance

A cut-off of 12 ppm based on the averaged upper prediction limit at 8 hours since smoking (Figure 3A; Y = 3.9-0.16×) for classifying recent smokers was validated using ROC curves (Figure 3B). At a cut-off of 12 ppm the average sensitivity was 90% and the specificity 94% for classifying recent smokers from smokers who had abstained from smoking for at least 8 hours. The robustness of the model was evaluated through 6 ROC curves in which different parts of the dataset had been left out. AUC for these were 0.98, 0.99, 0.91, 0.92, 0.96 and 0.94. In addition to the above generic cut-off, we tested individualised cut-off values based on the averaged upper prediction limit, but by utilizing also one previous measurement from each individual. The aim of this was to also consider the differences in Y0 values due to differences in smoking habits among the participants. This resulted in a model with a specificity of 95% and a sensitivity of 98% (Table S1).

## Discussion

In this study, we address an issue relevant to clinical and exploratory trials that include smoking subjects. With the aim to establish a method for assessing recent smoking status, we have evaluated CO_breath_ cut-off levels to classify recent smoking status. Specifically, a cut-off level of 12 ppm indicated whether a subject had smoked within the past 8 hours with a sensitivity of 90% and a specificity of 94%. The method was applied in our clinic at the Karolinska University Hospital for validation of smoking status of smokers participating in a translational study on COPD.

A number of previous studies have shown that CO_breath_ levels can be used to classify smokers from non-smokers in clinical settings. In the present study we expanded on these principles by developing a method based on CO_breath_ decline over time to assess the time since last cigarette within a population of smokers. By using individual cut-off values, we were able to separate smokers who had refrained from smoking for at least 8 hours from those who had smoked within this time frame with a specificity of 95% and a sensitivity of 98%. However, as calculation of individual values is impractical in a clinical setting, and requires prior collection of at least two CO_breath_ time points from the subject, we also evaluated a generic cut-off value. Through a model based on 38 smokers, we found that a cut-off value of 12 ppm could discriminate between recent smokers and smokers that have refrained from smoking for >8 hrs with a specificity of 94% and a sensitivity of 90%. Given that our data show that the levels of CO_breath_ in smokers are higher in the morning as compared to the corresponding time points in the afternoon, the indicated cut-off is intended for measurements performed in the morning. Although based on relatively few observations our data suggests a faster decline during the day, which is consistent with the findings of others [16]. Consequently we suggest using a correction factor of 1.33 for measurements performed in the afternoon, resulting in a cut-off of 12 ppm for studies evaluating smoking cessation during daytime.

It is known that CO in breath can be confounded by many factors such as diet, physical exercise, inflammatory diseases and time of the day [16], [25]. In our study, both smokers and non-smoking controls had slightly higher CO_breath_ at lunchtime, presumably caused by dietary factors for which the CO detector is cross-sensitive. As CO is produced endogenously as well, particularly during oxidative stress and inflammation, the potentially confounding effects of inflammatory lung disorders such as COPD and asthma needs to be considered. A study based on patients with asthma and COPD suggested slightly higher cut-off values of 10–11 ppm [14] in classification of smokers from non-smokers. However, increased levels of CO in exhaled air is primarily associated with exacerbations of the diseases [21], and may not be relevant for study designs where subjects without recent exacerbations are enrolled. This was the case in our study design, and no significant difference in CO_breath_ was detected when comparing smokers with normal lung function and smokers with COPD (Figure 3A).

Eighty-five percent of CO in the body is bound to hemoglobin in circulating erythrocytes, and the majority of the remaining CO is bound to myoglobin in the muscles [16]. As such, the slight gender differences in CO_breath_ decline rate observed in group 2 may be due to differences in muscle mass resulting in differing CO storage compartment. If this was the case, the effects would be more pronounced at longer time point since smoking. This is consistent with our findings where significant differences in half-lives were observed first after 8 hours of abstinence, and could serve as an explanation as to why the differences observed in group 2 was not seen in group 1. The relation between CO_breath_ and time elapsed since smoking has previously been addressed by Leitch *et al*. They observed that CO decline rate depends on initial CO levels, which is consistent with a logarithmic decrease [6], but on average has a decline of 3.4 ppm/hour. By necessity, a generic cut-off is therefore crude as initial CO_breath_ varies a lot between individuals.

A limitation with the group 2 data set is the absence of available data points between 3–7 hours since smoking, as very few measurements were performed within that time span. This may bias the specificity and sensitivity calculations in both models, resulting in an exaggerated discriminating ability. For proper evaluation of the proposed cut-off values, additional measurements performed in the critical time range are required. Also, the model is based on the assumption that the participants are accurate in their self-estimation of time since smoking. Likewise, the fact that the data from group 2 is discontinuous in the sense that the measurements are not limited to one day represents a possible further limitation in the strength of the resulting model. However, these patterns in the data set are highly reflective of the corresponding clinical situation: Either the subject has smoked the morning of the investigation, i.e. within the past few hours of CO monitoring, or the subject has adhered to the instructions and refrained from smoking since the evening before the procedure, and thus refrained from smoking for at least 8 hours.

To conclude, we propose a cut-off for classification of recent smoking status that is higher (12 ppm CO) compared to previous methods for classification between smokers and non-smokers. The application of the method is in clinical studies where recent smoking status has an impact on the outcome.

## Supporting Information

Figure S1 **Breath CO as measured on 6 smokers and 7 non-smokers at 1-hour-intervals during one day (group 1).** After having refrained from smoking during the night (>8 hours), exhaled CO was measured in the morning. Smokers were allowed to smoke one cigarette and then asked to refrain from further smoking throughout the day. **A.** Breath CO decline of smokers. Generally, measurements performed in the morning (>8 hours since smoking; dotted line indicates smoking of one cigarette) were higher than the corresponding >8 hrs since smoking measurements in the afternoon. **B.** Normalization of diurnal differences in CO elimination rates. Plot showing the ratios between breath CO>8 hrs since smoking measured in the morning divided by breath CO>8 hrs since smoking measured in the afternoon (group 1). For calculating the ratios, we used measured values closest to 8 hours since smoking for each individual. The derived median ratio 1.33 was used as normalization factor in subsequent evaluations. The value in brackets was measured 3.45 hours since smoking, and was excluded from the calculations. **C.** Breath CO of smokers plotted against the time of day. **D.** Breath CO of non-smokers plotted against time of day.
(EPS)

Figure S2 **Breath CO decline (smokers, group 2) plotted against self-estimated time since last cigarette.** **A.** Breath CO decline plotted against self-estimated time since last cigarette. The recordings were not performed continuously during one day, but performed over separate days. **B.** CO_breath_ decline of smokers in terms of gender (group 2). Gender differences (indicated using bars with hook) were significant at the time interval 9±1 hrs (p<0.05), but not at any other time point. When comparing breath CO decline in terms of distinct time intervals (both men and women; indicated with bars), there were significant (p<0.01) differences between each time bin except from 0.25±0.25 to 1±0.5. * p-value<0.05. (Mann Whitney rank sum test).
(EPS)

Figure S3 **CO_breath_ 8 hours since smoking in relation to current smoking history and lung capacity, respectively.** **A.** Breath CO and current cigarette consumption. **B.** Breath CO at 8 hours since smoking and total lung capacity (TLC).
(EPS)

Table S1 **Evaluation of individual cut-offs as predicted from the equation Y = Y0-0.16× (derived from the averaged decline rate obtained from smokers with normal lung function, group 2).**
(PDF)
