# Supplementary material for: Assessing Recent Smoking Status by Measuring Exhaled Carbon Monoxide Levels
Source: PLoS One. 2011 Dec 16;6(12):e28864. doi: 10.1371/journal.pone.0028864 (PMC3241681; doi:10.1371/journal.pone.0028864)
Supplement: Table S1 — Evaluation of individual cut-offs as predicted from the equation Y = Y0-0.16× (derived from the averaged decline rate obtained from smokers with normal lung function, group 2). (PDF) [file pone.0028864.s004.pdf]

Table S1. Evaluation of individual cut-offs as predicted from the equation Y=Y0-0.16x (derived from the averaged decline rate obtained from smokers with normal lung function, group 2).

Cut-off: Y=Y0-0.159x+ 1 S.D.	
**Measurements classified using individualized cut-off (0-9hrs):**	
**True Negatives (TN; correctly classified using the equation)**	58
**False Positives (FP; miss-classified using the equation)**	3
**≤ 7 hrs measurements tested against individualized cut-off (value at 8 hrs):**	
**True Positives (TP)**	60
**False Negatives (FN)**	1
**Sensitivity (TP/(TP+FN))**	98%
**Specificity (TN/(TN+FP))**	95%

Y0 for each individual was estimated by extrapolating the individual decay curve back to time 0. The derived equation plus one standard deviation was then used as cut-off.
